# Parts and Percentage

**Published:** 1891-05

**Authors:** 


### Parts and Percentage.

The chemist is not an errorist and therefore we submit to him many of the details of our work on account of his pharmaceutical ability and his knowledge of the elementary forces. Little do we think how much we depend upon him until we analyze his work, and yet we should be conversant with the knowledge of, if not of the manipulative skill in compounding medicines. Our success in the application of medicaments may be truly dependent upon the quality of the preparation the chemist or pharmacist prepares. With a poor drug the most skillful dentist may not be able to attain the desired effect, and hence our dependence upon the chemist.

It is often a necessity to combine medicaments and this calls into requisition our knowledge of the composition of the articles to be combined. Combinations may result in solids, liquids or gases, or, interchangeably united, might produce one of the three conditions.

As we have mostly to do with liquid combinations, on looking over those used by the dentist, we find that they are largely solutions.

A solution, properly speaking, is a combination of a liquid with either a solid or a gas to form a hemogeneous liquid. If there be an excess of any one of the formative elements, then the remaining part is a mechanical admixture.

To unite these elements we must resort to weights, measures, or volumes. In making solutions there is usually a condensation or expansion of volume, or a rise or fall of temperature, though solutions without these conditions are undoubtedly common.

For the sake of accuracy in the preparation of most of our medicaments, the combination by weight or measure is mostly used. The solution of solids with liquids is performed by weighing each in order to attain certain proportions, which is given in parts or a percentage of parts.

In expressing parts, the whole number of parts to be expressed is generally dependent upon the amount of solid to be dissolved. If large, we usually express the whole number of parts as 10, 20,

30, 40, 50 or 100, where, as when small, the total number is usually expressed in the thousands; thus we say, one part in ten or a hundred which is a large proportion compared to one part in one thousand.

On the contrary, whilst in expressing by parts, decimal notation is the usual plan pursued, in expressing by percentage, a deviation from the counting by tens is often noticed. Here, too, the entire number of parts is one hundred, and that is understood without saying 5 per cent. of 100.

There is really no difference in the terms parts and percentage but their use is one of convenience under different circumstances, it is only the mode of division that is different.

In accepting parts and percentages as modes of division we must not overlook the mighty grain, as an initial factor when the division is by weight. Many are ignorant of the values in grain weight of ounces and pounds and their transformations into ounces and pints liquid measures. The differences in weights arises from arbitrary fixture of the standard. A fluid ounce of water at 60° F. according to the United States standard weights 455.6216 grains; the U. S. Dispensatory gives it 455.6944 grains and the British standard is 455.6910 grains. The average weight is 455.6690 grains. Thus we see how inaccuracies may occur by the best chemists even though the differences only extend into the thousanths of a grain.

We wish to say in conclusion that such drugs as cocaine, now in use, are often made up into solutions in a slip-shod way and and not enough care is taken to equalize the parts or obtain the correct peicentages. In such cases the danger from their use is apparent. W.

---

CHICAGO is to have a complete dental library. The Chicago Dental Society has given its library to the Newberry Library under an agreement that it shall have ample and suitable accommodations in the new building, and that sufficient money shall be spent to complete and keep it equipped with all new publications in the future. The Newberry Library has an ample endowment and this will doubtless be one of the best dental libraries in the world. Happy Chicago.—H.

